# Identification of Distinct *Bacillus thuringiensis* 4A4 Nematicidal Factors Using the Model Nematodes *Pristionchus pacificus* and *Caenorhabditis elegans*

**DOI:** 10.3390/toxins6072050

**Published:** 2014-07-14

**Authors:** Igor Iatsenko, Angel Nikolov, Ralf J. Sommer

**Affiliations:** 1 Department for Evolutionary Biology, Max-Planck Institute for Developmental Biology, Spemannstrasse 37, 72076 Tuebingen, Germany; E-Mail: igor.iatsenko@tuebingen.mpg.de; 2 Institute for Chemistry and Biochemistry, Free University of Berlin, Thielallee 63, 14195 Berlin, Germany; E-Mail: angelnikolov17@yahoo.com

**Keywords:** *Bacillus thuringiensis*, *C. elegans*, *P. pacificus*, Cry toxins, Cry21Ha1, Vip1/Vip2, nematicidal factors, β-exotoxin

## Abstract

*Bacillus thuringiensis* has been extensively used for the biological control of insect pests. Nematicidal *B. thuringiensis* strains have also been identified; however, virulence factors of such strains are poorly investigated. Here, we describe virulence factors of the nematicidal *B. thuringiensis* 4A4 strain, using the model nematodes *Pristionchus pacificus* and *Caenorhabditis elegans*. We show that *B. thuringiensis* 4A4 kills both nematodes via intestinal damage. Whole genome sequencing of *B. thuringiensis* 4A4 identified Cry21Ha, Cry1Ba, Vip1/Vip2 and β-exotoxin as potential nematicidal factors. Only Cry21Ha showed toxicity to *C. elegans*, while neither Cry nor Vip toxins were active against *P. pacificus*, when expressed in *E. coli.* Purified crystals also failed to intoxicate *P. pacificus*, while autoclaved spore-crystal mixture of *B. thuringiensis* 4A4 retained toxicity, suggesting that primary β-exotoxin is responsible for *P. pacificus* killing. In support of this, we found that a β-exotoxin-deficient variant of *B. thuringiensis* 4A4, generated by plasmid curing lost virulence to the nematodes. Thus, using two model nematodes we revealed virulence factors of the nematicidal strain *B. thuringiensis* 4A4 and showed the multifactorial nature of its virulence.

## 1. Introduction

The Spore-forming bacterium *Bacillus thuringiensis* is successfully used for the biological control of agricultural pests [1]. Highly efficient, safe to humans and other vertebrates, completely biodegradable and strongly specific to the target hosts, *B. thuringiensis* is an important alternative to chemical insecticides [2,3,4,5,6,7,8]. *B. thuringiensis* relies on numerous virulence factors during the infection process [9]. Among them, Cry and Cyt toxins, produced as crystal inclusions during sporulation, play a major role in the killing of the host [3,4,5]. Most Cry and Cyt proteins belong to different classes of pore-forming toxins [4,5]. In contrast, some *B. thuringiensis* strains secrete toxins during vegetative growth, which are called vegetative insecticidal proteins (Vip) [10,11,12]. Another secreted toxin of *B. thuringiensis* is the thermostable secondary metabolite β-exotoxin, which has a wide range of insecticidal activity [13,14,15].

*B. thuringiensis* Cry and Cyt proteins are primarily toxic to a wide range of insect orders, but also to mites and protozoa [4,5]. Several families of nematicidal Cry proteins (Cry5, Cry6, Cry12, Cry13, Cry14, Cry21, Cry55) have also been described [16,17,18]. However, other nematicidal virulence factors of *B. thuringiensis* and the spectrum of their activity are poorly investigated. Very often the nematode *Caenorhabditis elegans* was used as the only model to study bacterial nematicidal factors and nematode defense mechanisms [16,18,19,20,21]. However, it is largely unknown how these mechanisms extend to other nematode species. The nematode *Pristionchus pacificus* has been extensively used for comparative studies with *C. elegans* [22], including innate immunity and its interactions with bacteria [23,24,25,26,27]. *P. pacificus* is resistant to pathogenic *Bacillus* spores [27], purified *B. thuringiensis* Cry5B toxin [16,28] and to *B. thuringiensis* DB27 [24], which produces three novel Cry21 toxins [18,29]. Given that *P. pacificus* is resistant to some Cry toxins that have been tested, *B. thuringiensis* strains pathogenic to *P. pacificus* will very likely rely on mechanisms distinct from Cry toxins to kill this nematode. Such mechanisms are difficult to identify using only nematodes that are susceptible to Cry toxins (like *C. elegans*). Thus, *P. pacificus* resistance to Cry toxins facilitates the use of this nematode to study *B. thuringiensis* nematicidal virulence factors distinct from Cry toxins.

To investigate whether there are *B. thuringiensis* strains that are toxic to *P. pacificus* and if so, what virulence factors they use, we exposed *P. pacificus* to several described *B. thuringiensis* strains. We identified *B. thuringiensis* 4A4 as the most virulent among six tested strains and characterized it further. *B. thuringiensis* 4A4 causes extensive intestinal damage in *P. pacificus* and *C. elegans*. Whole genome sequencing revealed several potential nematicidal factors of *B. thuringiensis* 4A4, including Cry21Ha, Cry1Ba and Vip1/Vip2 toxins. Functional characterization of these toxins suggests a multifactorial nature of *B. thuringiensis* 4A4 virulence to nematodes.

## 2. Results and Discussion

### 2.1. B. thuringiensis 4A4 Is Virulent to P. pacificus

**Figure 1:**
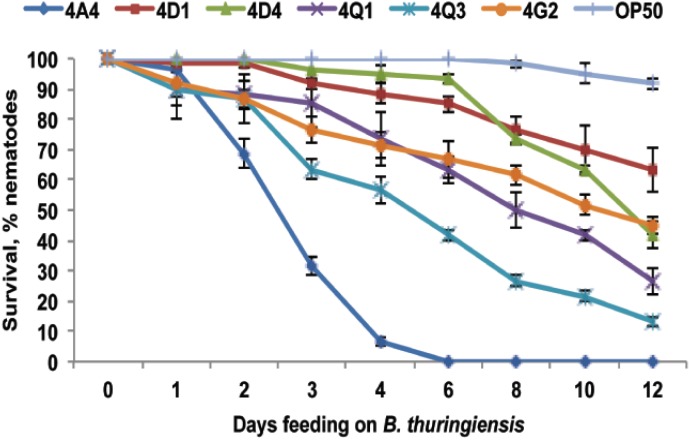
*P. pacificus* survival on different *B. thuringiensis* strains. For survival curves, percent of surviving nematodes (% nematodes) is plotted as a function of time. The data shown are means ± SEM.

To identify *B. thuringiensis* strains pathogenic to *P. pacificus*, we exposed this nematode to several representative strains of different *B. thuringiensis* serovars and scored survival. As shown in Figure 1, *B. thuringiensis* strains strongly differ in their ability to kill *P. pacificus*. *B. thuringiensis* subsp. *israelensis* (4Q1 and 4Q3) showed moderate toxicity to *P. pacificus*, while *B. thuringiensis* subsp. *kurstaki* (4D1 and 4D4) exhibited very mild effect on *P. pacificus* survival. Among the tested strains *B. thuringiensis* subsp. *thuringiensis* (4A4) exhibited the strongest effect and was chosen for further analysis. This strain was not characterized before and the spectrum of its activity is not known.

### 2.2. B. thuringiensis 4A4 Damages Intestines of P. pacificus and C. elegans

**Figure 2:**
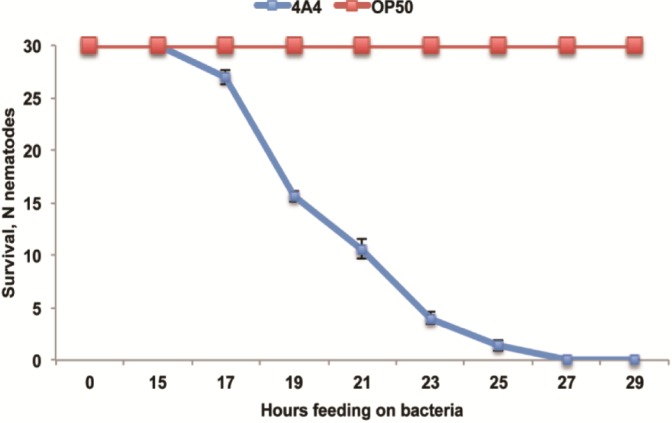
*C. elegans* survival on *B. thuringiensis* 4A4. *E. coli* OP50 is used as a control. Number of surviving nematodes (N nematodes) is plotted as a function of time. The data shown are means ± SEM.

*B. thuringiensis* 4A4 is also toxic to *C. elegans*. As shown in Figure 2, virulence of *B. thuringiensis* 4A4 against *C. elegans* is fast and worms are completely killed within 25 h. In contrast, *P. pacificus* tolerates *B. thuringiensis* 4A4 for several days and only after four days more than 90% of the worms are dead (Figure 1). These results are in agreement with previous studies, which showed that *P. pacificus* is generally more resistant to pathogens than *C. elegans* [23,24,25,26,27].

**Figure 3:**
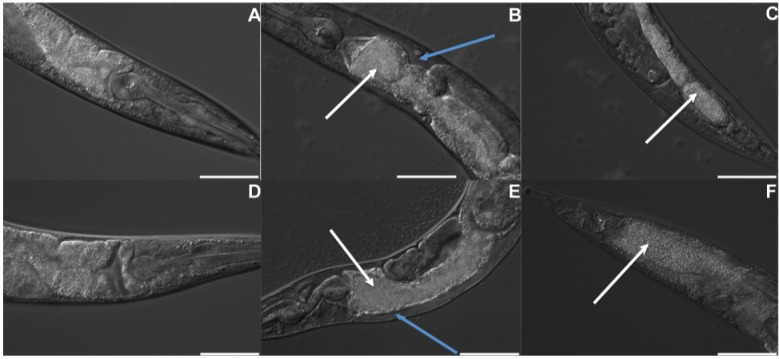
Intestinal changes in *B. thuringiensis* 4A4-infected worms. *C. elegans* (**A**) and *P. pacificus* (**D**) on *E. coli* OP50. *B. thuringiensis* 4A4 accumulates (white arrows) and causes shrinkage (blue arrows) and damage of anterior (*C. elegans* (**B**), *P. pacificus* (**E**)) and posterior parts (*C. elegans* (**C**), *P. pacificus* (**F**)) of the intestine. Scale bar is 50 μm.

In the next set of experiments, we used both nematodes to further characterize virulence and to elucidate virulence factors of *B. thuringiensis* 4A4. First, we examined infected worms microscopically. We noticed that compared to control worms on *E. coli* OP50 (Figure 3A,D), *B. thuringiensis* 4A4-infected worms exhibited intestinal destructive changes (Figure 3). For both, *C. elegans* and *P. pacificus*, we observed intestinal shrinkage, dissociation from body walls and accumulation of bacteria in the intestine (Figure 3). Similar changes were reported for *C. elegans* worms exposed to *B. thuringiensis* DB27 [18] and to Cry5B toxin [30], indicating that *B. thuringiensis* 4A4 might use similar virulence mechanism. Thus, *P. pacificus*, like *C. elegans*, shows typical intoxication phenotypes, although this phenomenon was only seen after much longer exposure time.

### 2.3. B. thuringiensis 4A4 Is Avoided by Nematodes

**Figure 4:**
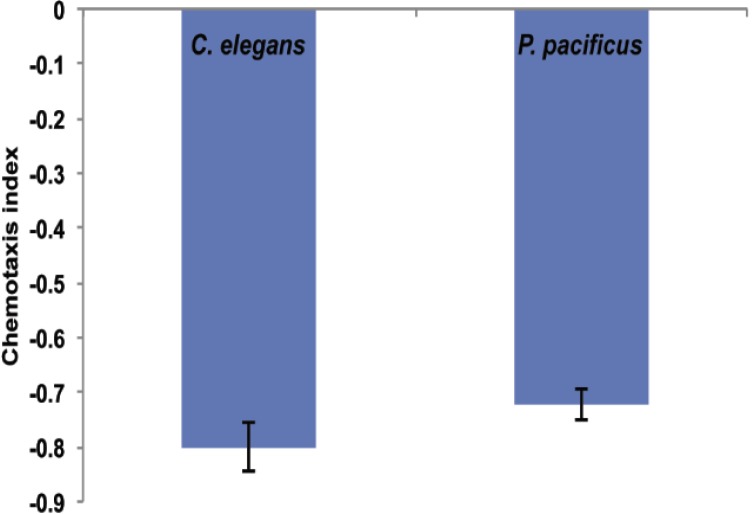
Chemotaxis experiment (4A4 *vs.* OP50). Negative chemotaxis index indicates that nematodes avoided 4A4 and mostly moved to OP50 spot. Results were scored after 16 h. The data shown are means ± SEM.

Pathogen avoidance is a behavioral defense mechanism that *C. elegans* uses against several pathogens [31,32,33]. In agreement with this, we found that both, *C. elegans* and *P. pacificus* strongly avoided *B. thuringiensis* 4A4 in chemotaxis assays (Figure 4A), preferentially moving towards an *E. coli* OP50 spot. A few worms that initially moved to and came into contact with 4A4, then migrated to OP50. These results suggest that 4A4 is either a low quality food for nematodes or is toxic to nematodes.

### 2.4. B. thuringiensis 4A4 Cry Toxins Are Involved in Nematode Killing

**Figure 5:**
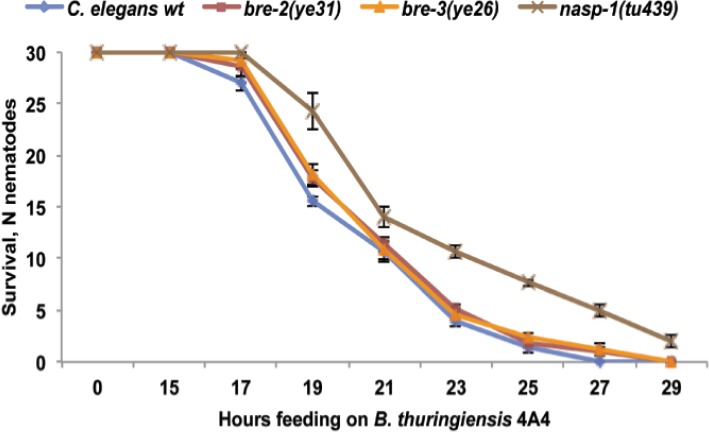
Survival of *C. elegans* wild-type, *bre* mutants and *nasp-1* mutant on *B. thuringiensis* 4A4. Number of surviving nematodes (N nematodes) is plotted as a function of time. *nasp-1* mutants showed a significantly (*p* = 0.006) increased survival rate compared to wild-type and *bre* mutants. Survival of *bre* mutants is not different from wild-type (*p* > 0.05). The data shown are means ± SEM.

To test whether 4A4 relies on Cry toxins and whether these Cry toxins act similar to nematicidal Cry5B and Cry21 toxins, we exposed *C. elegans bre* mutants (resistant to Cry5B toxin [34]) and the *nasp-1* mutant (resistant to *B. thuringiensis* DB27 and Cry21 toxins [18,21]) to *B. thuringiensis* 4A4. As shown in Figure 5, *bre* mutants are as susceptible to *B. thuringiensis* 4A4 as wild-type worms, suggesting that 4A4 uses distinct from Cry5B mechanism to target nematodes, while *nasp-1* mutant animals are more resistant. These findings suggest that *B. thuringiensis* 4A4 toxins might act similarly to Cry21 toxins, but differently than Cry5B toxin.

### 2.5. Identification of Potential Toxins

Whole genome sequencing has proven to be a useful and efficient tool for the discovery and identification of *B. thuringiensis* toxins [29,35]. To identify Cry toxins produced by *B. thuringiensis* 4A4, we sequenced its genome (manuscript in preparation). Analysis of the genome sequence using BtToxin scanner [35] revealed that this strain harbors several toxins with potential activity against nematodes. Specifically, we identified three candidate genes that showed 100% sequence similarity to previously described Cry1Ba1, Cry21Ha1 and Vip1/Vip2 genes Among these toxins, the recently discovered Cry21Ha1 was shown to have activity against *C. elegans* [18]. However, neither Cry1Ba nor Vip1/Vip2 toxins have been tested previously for their potential nematicidal activity.

**Figure 6:**
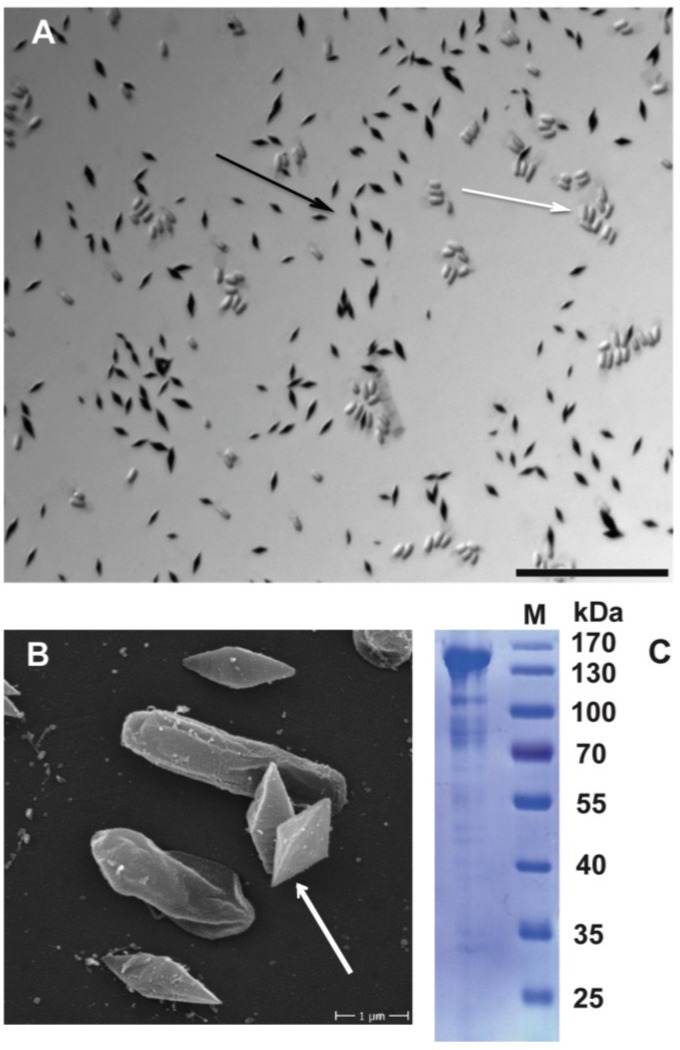
*B. thuringiensis* 4A4 produces crystal proteins. (**A)** Light microscopy image of Coomassie-stained spore-crystal mixture of *B. thuringiensis* 4A4. Spores are unstained (white arrow), black structures are crystal proteins (black arrow). Scale bar is 20 μm; (**B**) Scanning electron microscopy image of spore-crystal mixture of *B. thuringiensis* 4A4. Bi-pyramidal crystals are shown (pointed with white arrow); (**C**) SDS-PAGE profile of *B. thuringiensis* 4A4 crystal proteins. Dominant protein of around 140 kDa is shown.

Using light microscopy and Coomassie staining of spore-crystal mixtures, we confirmed that *B. thuringiensis* 4A4 indeed produces Cry proteins, which are shown as stained crystals in Figure 6A. In addition, scanning electron microscopy revealed a bi-pyramidal shape of the crystals (Figure 6B). Cry1 proteins very often form bi-pyramidal crystals, which is consistent with the presence of the Cry1Ba gene in the 4A4 genome. These crystals are composed of a major protoxin of 140 kDa as shown in Figure 6C. Based on the translated sequence, it is very likely that the 140 kDa band is formed by the Cry21Ha protein, since the size of Cry1Ba protein should be around 135 kDa according to the translated sequence.

**Figure 7:**
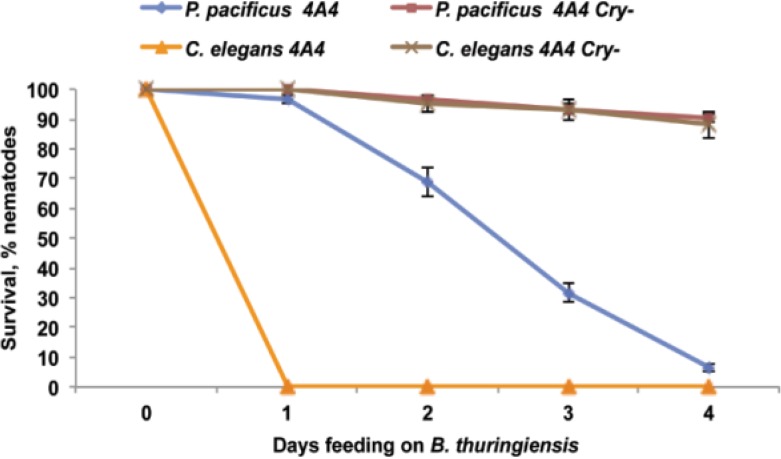
*P. pacificus* and *C. elegans* survival on *B. thuringiensis* 4A4 wild-type (4A4) and crystal-negative (Cry-) derivative. Cry- is impaired in ability to kill both nematodes. The data shown are means ± SEM.

To determine if Cry toxins are indeed necessary for nematode killing, we generated an acrystaliferous variant of *B. thuringiensis* 4A4 by plasmid curing. This variant is strongly impaired in the ability to kill nematodes (Figure 7), suggesting that Cry toxins of *B. thuringiensis* 4A4 may play a major role in the virulence to nematodes.

### 2.6. Functional Validation of Toxins

**Figure 8:**
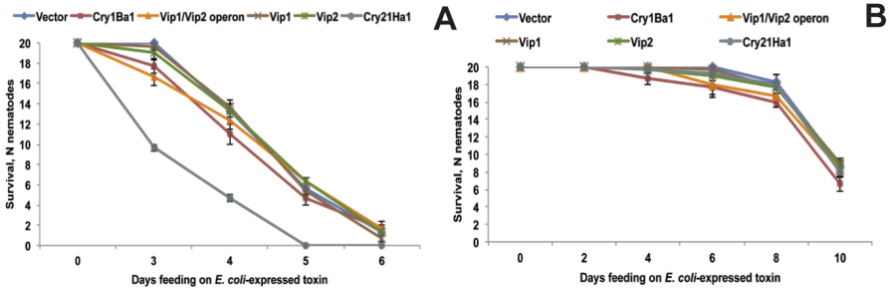
*C. elegans* (**A**) and *P. pacificus* (**B**) survival on *E. coli* clones that express different toxins. Only Cry21Ha (**A**) significantly (*p* > 0.001) reduces *C. elegans* survival compared to vector control. No differences are observed between toxin and vector treatments in case of *P. pacificus*. The data shown are means ± SEM.

To test if the identified toxins are indeed sufficient for nematode killing, they were expressed in *E. coli* and fed to *C. elegans* and *P. pacificus*. As shown in Figure 8A, *C. elegans* dies when fed an *E. coli* clone that expresses Cry21Ha toxin. Also, *C. elegans* survival on Cry1Ba toxin was slightly (but not significantly) reduced compared to vector control (Figure 8A). In contrast, Vip1 and Vip2 toxins expressed individually or as operon had no effect on *C. elegans* survival (Figure 8A), suggesting that these toxins do not have activity against *C. elegans*. In the case of *P. pacificus*, we did not observe any differences between toxin and control treatments (Figure 8B). This result indicates that all tested proteins are not active against *P. pacificus* as single toxins. Very likely combinations of these factors and/or addition of other factors are required for toxicity. Therefore, we also exposed both nematodes to different combinations of the three toxins. In case of *C. elegans*, even when the three toxins were combined, mortality was not increased compared to what was observed for exposition to the single Cry21Ha. *P. pacificus* also remained unaffected when exposed to combinations of toxins (not shown).

**Figure 9:**
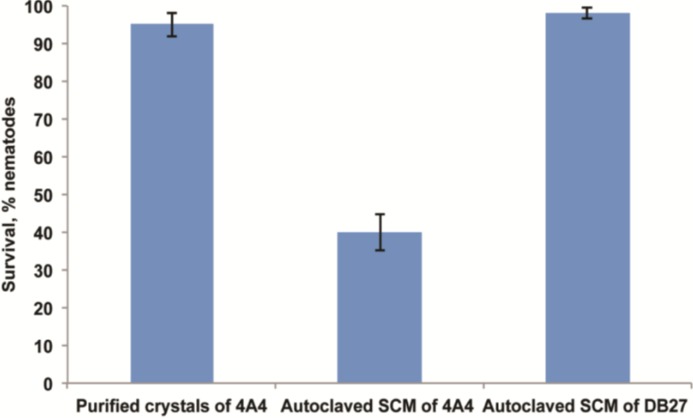
*P. pacificus* survival after 5 days exposure to purified crystals or autoclaved spore-crystal mixture (SCM) of 4A4. *P. pacificus* survival is significantly (*p* < 0.0001) reduced on SCM of 4A4 compared to SCM of DB27, which is not toxic and does not produce β-exotoxin. The data shown are means ± SEM.

The lack of a grinder in *P. pacificus* was suggested to be the potential reason for the resistance to *E. coli*-expressed Cry toxins [16]. Since *E. coli* cells are not mechanically lysed in *P. pacificus* given the absence of the grinder, like it is the case in *C. elegans*, worms might never be exposed to the toxins. To rule out this possibility, we exposed *P. pacificus* to purified crystals. However, even at concentration above 250 μg/mL we did not observe any detrimental effect on the health of the worm (Figure 9), which suggests that Cry toxins produced by 4A4 are not involved in *P. pacificus* killing. However, our finding that an acrystaliferous, plasmid-cured variant of *B. thuringiensis* 4A4 is impaired in its ability to kill *P. pacificus*, indicates that a plasmid-encoded factor is essential for *P. pacificus* killing. *B. thuringiensis* subsp. *thuringiensis* strains (like 4A4) that contain Cry1 toxins very often also produce plasmid-encoded β-exotoxin [13,14,15]. Analysis of the 4A4 genome revealed that this strain indeed has all genes necessary for β-exotoxin production, all of which are located on a plasmid right next to Cry1Ba1. Given the wide spectrum of β-exotoxin insecticidal activity, it is possible that it also acts against nematodes. In support of this, we noticed that autoclaved crude spore-crystal mixture of 4A4 was toxic to *C. elegans* and *P. pacificus* (Figure 9). These results strongly support the hypothesis that β-exotoxin, and not Cry proteins, is responsible for 4A4 toxicity to *P. pacificus*, while both Cry21Ha and β-exotoxin are active against *C. elegans*. Taken together, using two model nematodes, we identified multifactorial nature of *B. thuringiensis* 4A4 virulence mechanism to nematodes, with the crucial role of Cry toxins and β-exotoxin.

## 3. Experimental Section

### 3.1. Nematode and Bacterial Strains

The following strains were provided by the *Caenorhabditis* Genetics Center (University of Minnesota, Minneapolis, MN, USA): *C. elegans* wild-type Bristol (N2), *bre-2*(*ye31*), *bre-3*(*ye26*). *P. pacificus* PS312 wild type strain is a derivative of an isolate from Pasadena, California [36]. Nematodes were maintained on Nematode Growing Media (NGM) agar plates with *E. coli* OP50 as a food source and stored at 20 °C. *B. thuringiensis* DB27 was isolated by our group [24]. The following *B. thuringiensis* strains were obtained from *Bacillus* Genetic Stock Center: *B. thuringiensis* subsp. *galleriae* (4G2), *B. thuringiensis* subsp. *thuringiensis* (4A4), *B. thuringiensis* subsp. *kurstaki* (4D1), *B. thuringiensis* subsp. *kurstaki* (4D4), *B. thuringiensis* subsp. *israelensis* (4Q1), *B. thuringiensis* subsp. *Israelensis* (4Q3). Acrystaliferous derivative of *B. thuringiensis* 4A4 was generated is this study by plasmid curing as described previously [18].

### 3.2. Nematode Killing Assays

*B. thuringiensis* strains were grown overnight in a shaking incubator at 30 °C in Luria-Bertani (LB) broth. Then, 80 μL of the culture were spread to the edges of 6 cm NGM plates and plates were incubated for 5–7 days at 25 °C before the assay. We noticed that the incubation time strongly affects the toxicity of bacteria, probably due to accumulation of different amount of toxins. Therefore, 5–7 days incubation period was chosen to ensure full sporulation of bacteria. Adult worms (20–30) were put per plate in three to six independent replicates and were monitored for survival. Survival assays were repeated multiple times and conducted at 25 °C.

### 3.3. Chemotaxis Assay

Chemotaxis assays were modified from previous studies [23]. Briefly, overnight suspension of *B. thuringiensis* 4A4 (25 μL) was placed 0.5 cm away from the edge of a 6 cm Petri dish filled with NGM medium. The same amount of *E. coli* OP50 was placed on the opposing side and acted as the counter attractant. Plates were incubated for 24 h before the assay. Approximately 50–200 J4/adult stage nematodes were placed between the two bacterial spots. All nematodes used were previously fed on *E. coli* OP50. Plates were then sealed with Parafilm^®^ (Bemis Flexible Packaging, Neenah, WI, USA) and stored at room temperature in the dark. After defined periods, the number of nematodes found in each bacterial spot was recorded. A chemotaxis index was used to score the response of the nematodes, which consisted of: number of nematodes in the test bacteria—numbers of nematodes in control bacteria/total number of nematodes counted [23]. This gave a chemotaxis score ranging from −1.0 (total repulsion from test bacteria) to 1.0 (total attraction towards test bacteria). A score of around 0 means there were equal numbers of nematodes in each bacterial spot. Five plates were used per replicate, and the procedure was repeated five times.

### 3.4. Coomassie Stain and Electron Microscopy of Crystals

*B. thuringiensis* 4A4 spore-crystal mixtures (1 mL of liquid culture) were collected by centrifugation, washed three times with 1 M NaCl and ice-cold distilled water. The washed spore-crystal mixtures were re-suspended in 1 mL of distilled water, and 10 μL of 2-fold diluted sample was dropped onto a glass slide. Samples were fixed in 1% OsO_4_, air-dried overnight and then coated with gold. The SEM observation was conducted on a HITACHI S-800 (Hitachi, Tokyo, Japan) following instructions for the device. For light microscopy, spore-crystal mixtures were spread on a glass slide, heat-fixed, stained with Coomassie blue (0.133% Coomassie blue in 50% acetic acid) [37] and observed under 100× magnification using immersion oil.

### 3.5. Solubilization and SDS-PAGE Profiling of Crystal Proteins

Spore-crystal mixtures were collected and washed as described above. The spore-crystal pellet was resuspended in solubilization buffer (50 mM Na_2_CO_3_, 25 mM DTT, pH 10.5) and incubated at 37 °C for two hours. Insoluble remainings were removed by centrifugation and the solubilized proteins from the supernatant were analyzed using SDS-PAGE.

### 3.6. Toxin Cloning, Protein Expression and Killing Assay

The toxin genes were PCR amplified from genomic DNA of *B. thuringiensis* 4A4, digested with BamHI restriction enzyme and ligated into the BamHI site of the expression vector pET-22b. Recombinant plasmids were electroporated into *E. coli* BL21. Bacteria were grown at 37 °C to midlog phase (OD_600_ = 0.6–0.8), and expression of proteins was induced with 1 mM isopropyl β-D-1-thiogalactopyranoside (IPTG). After induction, bacteria were grown at 30 °C for 4 h and then 30 μL of bacterial culture were spread in the center of Enriched Nematode Growth (ENG) plates [38], containing 1 mM IPTG (ENG-IC plates). Plates were incubated overnight at 25 °C and then used for nematode toxicity assays. Synchronized nematodes at adult stage (20 per plate) were transferred to toxicity plates and monitored for survival and intoxication. Nematodes were transferred to fresh plates every day and were considered dead when they failed to respond to touch. *E. coli* BL21 transformed with empty pET-22b vector was used as a control.

### 3.7. Effect of Purified Crystals and SCM on Survival

Crystals of 4A4 were purified using sucrose gradient as described previously [20]. Spore-crystal mixtures were prepared as described previously [39]. Autoclaved SCM was prepared in the following way: bacteria were grown in sporulation medium for one week and the obtained mixture of spores and crystals was autoclaved together with the growth medium. Spores and crystals were spun down and used at defined concentration in singe well assays. Single well assay [30,38] was used to test the effect of purified crystals and spore-crystal mixtures on worms’ survival. Briefly, single nematodes were picked in individual wells of 96-well plate. Each well contained: *S*-medium, defined concentration of purified crystals or spore-crystal mixture, *E. coli* OP50 as a food source, tetracycline and chloramphenicol (30 μg each) to prevent spore germination and bacterial growth. Survival of nematodes was scored after 5 days.

### 3.8. Statistical Analysis

Kaplan-Meier non-parametric comparison and a log-rank test (Minitab version 14, Minitab Inc., State College, PA, USA, 2004) were used for statistical analysis of survival curves based on the number of survivors at the sampled time points. In cases where multiple replicates were examined in one experiment, the average survival rate at each time point was determined. Bonferroni correction was applied when multiple comparisons were performed. Student’s *t*-tests were used for comparison of two treatments. Statistical significance was set at *p* ≤ 0.05.

## 4. Conclusions

*Bacillus thuringiensis* and its toxins have been extensively studied, primarily in terms of their toxicity to insect hosts. Nematode-specific *Bacillus thuringiensis* strains have also been identified, however, virulence factors of these strains are poorly investigated. Additionally, the nematode *C. elegans* very often was used as the only model to study nematicidal properties of *Bacillus thuringiensis*. Here, in addition to *C. elegans*, we used another model nematode *Pristionchus pacificus* to identify nematicidal factors of *Bacillus thuringiensis* 4A4. This strain kills both nematodes via intestinal damage. With the help of whole genome sequencing we identified Cry toxins (Cry1Ba and Cry21Ha), binary Vip1/Vip2 toxin and β-exotoxin as potential virulence factors. When Cry and Vip toxins were expressed in *E. coli*, only Cry21Ha showed toxicity to *C. elegans*, while none of these toxins was active against *P. pacificus*. Additionally, purified crystals failed to kill *P. pacificus*, while crude spore-crystal mixture retained toxicity to *P. pacificus* even after autoclaving, suggesting that β-exotoxin is primarily responsible for *P. pacificus* killing. Thus, our study not only reveals nematicidal factors of *B. thuringiensis* 4A4 and the multifactorial nature of its virulence, but also suggests that *B. thuringiensis* relies on different virulence factors to infect different nematode hosts. These results prove the importance of using multiple hosts in the studies of *B. thuringiensis* virulence mechanisms.

## References

[1] Bravo A., Likitvivatanavong S., Gill S.S., Soberón M. (2011). *Bacillus thuringiensis*: A story of a successful bioinsecticide. Insect Biochem. Mol. Biol..

[2] Bravo A., Gómez I., Porta H., García-Gómez B.I., Rodriguez-Almazan C., Pardo L., Soberón M. (2013). Evolution of *Bacillus thuringiensis* Cry toxins insecticidal activity. Microb. Biotechnol..

[3] Pardo-López L., Soberón M., Bravo A. (2013). *Bacillus thuringiensis* insecticidal three-domain Cry toxins: Mode of action, insect resistance and consequences for crop protection. FEMS Microbiol. Rev..

[4] Bravo A., Gill S.S., Soberón M. (2007). Mode of action of *Bacillus thuringiensis* Cry and Cyt toxins and their potential for insect control. Toxicon.

[5] Soberón M., López-Díaz J.A., Bravo A. (2013). Cyt toxins produced by *Bacillus thuringiensis*: A protein fold conserved in several pathogenic microorganisms. Peptides.

[6] Soberón M., Pardo L., Muñóz-Garay C., Sánchez J., Gómez I., Porta H., Bravo A. (2010). Pore formation by Cry toxins. Adv. Exp. Med. Biol..

[7] De Maagd R.A., Bravo A., Crickmore N. (2001). How *Bacillus thuringiensis* has evolved specific toxins to colonize the insect world. Trends Genet..

[8] Ben-Dov E. (2014). *Bacillus thuringiensis* subsp. *israelensis* and its dipteran-specific toxins. Toxins.

[9] Raymond B., Johnston P.R., Nielsen-LeRoux C., Lereclus D., Crickmore N. (2010). *Bacillus thuringiensis*: An impotent pathogen?. Trends Microbiol..

[10] Hernández-Rodríguez C.S., Boets A., van Rie J., Ferré J. (2009). Screening and identification of vip genes in *Bacillus thuringiensis* strains. J. Appl. Microbiol..

[11] Estruch J.J., Warren G.W., Mullins M.A., Nye G.J., Craig J.A., Koziel M.G. (1996). Vip3A, a novel *Bacillus thuringiensis* vegetative insecticidal protein with a wide spectrum of activities against lepidopteran insects. Proc. Natl. Acad. Sci. USA.

[12] Warren G.W., Carozzi N., Koziel M. (1997). Vegetative insecticidal proteins: Novel proteins for control of corn pests. Advances in Insect Control: The Role of Transgenic Plants.

[13] Espinasse S., Gohar M., Chaufaux J., Buisson C., Perchat S., Sanchis V., Espinasse S., Gohar M., Chaufaux J., Buisson C. (2002). Correspondence of high levels of β-exotoxin I and the presence of cry1B in *Bacillus thuringiensis* correspondence of high levels of β-Exotoxin I and the presence of cry1B in *Bacillus thuringiensis*. Appl. Environ. Microbiol..

[14] Liu X.-Y., Ruan L.-F., Hu Z.-F., Peng D.-H., Cao S.-Y., Yu Z.-N., Liu Y., Zheng J.-S., Sun M. (2010). Genome-wide screening reveals the genetic determinants of an antibiotic insecticide in *Bacillus thuringiensis*. J. Biol. Chem..

[15] Sara Hernández C., Martínez C., Porcar M., Caballero P., Ferré J. (2003). Correlation between serovars of *Bacillus thuringiensis* and type I β-exotoxin production. J. Invertebr. Pathol..

[16] Wei J.-Z., Hale K., Carta L., Platzer E., Wong C., Fang S.-C., Aroian R.V. (2003). *Bacillus thuringiensis* crystal proteins that target nematodes. Proc. Natl. Acad. Sci. USA.

[17] Guo S., Liu M., Peng D., Ji S., Wang P., Yu Z., Sun M. (2008). New strategy for isolating novel nematicidal crystal protein genes from *Bacillus thuringiensis* strain YBT-1518. Appl. Environ. Microbiol..

[18] Iatsenko I., Boichenko I., Sommer R.J. (2014). *Bacillus thuringiensis* DB27 produces two novel protoxins, Cry21Fa1 and Cry21Ha1, which act synergistically against nematodes. Appl. Environ. Microbiol..

[19] Kao C.-Y., Los F.C.O., Huffman D.L., Wachi S., Kloft N., Husmann M., Karabrahimi V., Schwartz J.-L., Bellier A., Ha C. (2011). Global functional analyses of cellular responses to pore-forming toxins. PLoS Pathog..

[20] Luo X., Chen L., Huang Q., Zheng J., Zhou W., Peng D., Ruan L., Sun M. (2013). *Bacillus thuringiensis* metalloproteinase Bmp1 functions as a nematicidal virulence factor. Appl. Environ. Microbiol..

[21] Iatsenko I., Sinha A., Rödelsperger C., Sommer R.J. (2013). New role for DCR-1/dicer in *Caenorhabditis elegans* innate immunity against the highly virulent bacterium *Bacillus thuringiensis* DB27. Infect. Immun..

[22] Sommer R.J., McGaughran A. (2013). The nematode *Pristionchus pacificus* as a model system for integrative studies in evolutionary biology. Mol. Ecol..

[23] Rae R., Riebesell M., Dinkelacker I., Wang Q., Herrmann M., Weller A.M., Dieterich C., Sommer R.J. (2008). Isolation of naturally associated bacteria of necromenic *Pristionchus* nematodes and fitness consequences. J. Exp. Biol..

[24] Rae R., Iatsenko I., Witte H., Sommer R.J. (2010). A subset of naturally isolated *Bacillus* strains show extreme virulence to the free-living nematodes *Caenorhabditis elegans* and *Pristionchus pacificus*. Environ. Microbiol..

[25] Sinha A., Rae R., Iatsenko I., Sommer R.J. (2012). System wide analysis of the evolution of innate immunity in the nematode model species *Caenorhabditis elegans* and *Pristionchus pacificus*. PLoS One.

[26] Rae R., Sinha A., Sommer R.J. (2012). Genome-wide analysis of germline signaling genes regulating longevity and innate immunity in the nematode *Pristionchus pacificus*. PLoS Pathog..

[27] Rae R., Witte H., Rödelsperger C., Sommer R.J. (2012). The importance of being regular: *Caenorhabditis elegans* and *Pristionchus pacificus* defecation mutants are hypersusceptible to bacterial pathogens. Int. J. Parasitol..

[28] Hui F., Scheib U., Hu Y., Sommer R.J., Aroian R.V., Ghosh P. (2012). Structure and Glycolipid Binding Properties of the Nematicidal Protein Cry5B. Biochemistry.

[29] Iatsenko I., Corton C., Pickard D.J., Dougan G., Sommer R.J. (2014). Draft Genome Sequence of Highly Nematicidal *Bacillus thuringiensis* DB27. Genome Announc..

[30] Marroquin L.D., Elyassnia D., Griffitts J.S., Feitelson J.S., Aroian R.V. (2000). *Bacillus thuringiensis* (Bt) toxin susceptibility and isolation of resistance mutants in the nematode *Caenorhabditis elegans*. Genetics.

[31] Schulenburg H., Ewbank J.J. (2007). The genetics of pathogen avoidance in *Caenorhabditis elegans*. Mol. Microbiol..

[32] Hasshoff M., Böhnisch C., Tonn D., Hasert B., Schulenburg H. (2007). The role of *Caenorhabditis elegans* insulin-like signaling in the behavioral avoidance of pathogenic *Bacillus thuringiensis*. FASEB J..

[33] Zhang Y., Lu H., Bargmann C.I. (2005). Pathogenic bacteria induce aversive olfactory learning in *Caenorhabditis elegans*. Nature.

[34] Griffitts J.S., Haslam S.M., Yang T., Garczynski S.F., Mulloy B., Morris H., Cremer P.S., Dell A., Adang M.J., Aroian R.V. (2005). Glycolipids as receptors for *Bacillus thuringiensis* crystal toxin. Science.

[35] Ye W., Zhu L., Liu Y., Crickmore N., Peng D., Ruan L., Sun M. (2012). Mining new crystal protein genes from *Bacillus thuringiensis* on the basis of mixed plasmid-enriched genome sequencing and a computational pipeline. Appl. Environ. Microbiol..

[36] Sommer R.J., Sternberg P.W. (1996). Apoptosis and change of competence limit the size of the vulva equivalence group in *Pristionchus pacificus*: A genetic analysis. Curr. Biol..

[37] Rampersad J., Khan A., Ammons D. (2002). Usefulness of staining parasporal bodies when screening for *Bacillus thuringiensis*. J. Invertebr. Pathol..

[38] Bischof L.J., Huffman D.L., Aroian R.V. (2006). Assays for toxicity studies in *C. elegans* with Bt crystal proteins. Methods Mol. Biol..

[39] Borgonie G., Claeys M., Leyns F., Arnaut G., DeWaele D., Coomans A.V. (1996). Effect of nematicidal *Bacillus thuringiensis* strains on free-living nematodes 1. Light microscopic observations, species and biological stage specificity and identification of resistant mutants of *Caenorhabditis elegans*. Nematology.

